# Synthesis and physicochemical characterization of novel phenotypic probes targeting the nuclear factor-kappa B signaling pathway

**DOI:** 10.3762/bjoc.9.103

**Published:** 2013-05-08

**Authors:** Paul M Hershberger, Satyamaheshwar Peddibhotla, E Hampton Sessions, Daniela B Divlianska, Ricardo G Correa, Anthony B Pinkerton, John C Reed, Gregory P Roth

**Affiliations:** 1 Conrad Prebys Center for Chemical Genomics, Sanford-Burnham Medical Research Institute at Lake Nona, 6400 Sanger Road Orlando, FL 32827, USA; 2 Sanford-Burnham Medical Research Institute, 10901 North Torrey Pines Road, La Jolla, CA 92037, USA

**Keywords:** ML029, ML130, ML146, ML236, ML237

## Abstract

Activation of nuclear factor-kappa B (NF-κB) and related upstream signal transduction pathways have long been associated with the pathogenesis of a variety of inflammatory diseases and has recently been implicated in the onset of cancer. This report provides a synthetic and compound-based property summary of five pathway-related small-molecule chemical probes identified and optimized within the National Institutes of Health-Molecular Libraries Probe Center Network (NIH-MLPCN) initiative. The chemical probes discussed herein represent first-in-class, non-kinase-based modulators of the NF-κB signaling pathway, which were identified and optimized through either cellular phenotypic or specific protein-target-based screening strategies. Accordingly, the resulting new chemical probes may allow for better fundamental understanding of this highly complex biochemical signaling network and could advance future therapeutic translation toward the clinical setting.

## Introduction

The Molecular Libraries Network was established in 2005 as an academic pilot-scale (MLSCN) screening effort for exploring potential new therapeutic targets stemming from the human genome project. After initial successes in high-throughput chemical screening (HTS), efforts in medicinal chemistry and exploratory pharmacology were added in order to leverage and advance the small-molecule compounds discovered within the HTS process. The program then advanced a broader probe-production initiative (MLPCN) in 2008. This consortium resulted in the harnessing of chemical biology resources from four comprehensive centers and five specialty centers, which focused on either medicinal chemistry or a specific technical screening capability as an overall academic, collaborative scientific network [1]. In general terms, the overall vision and NIH mission encompasses the discovery of unique chemical tools that will be useful to interested scientific investigators to assist them with advancing basic in vitro and in vivo studies for testing new hypotheses in disease modulation.

Within this report, the outcomes of two Sanford-Burnham projects for chemical probe discovery are discussed, highlighting five examples of thematic small-molecule chemical probes discovered through our center’s efforts. Within the realm of immunology and inflammation research, many cellular pathways leading to the activation of NF-κB family of transcription factors have been identified and several excellent reviews are available [2–5]. In general, these pathways have been shown to participate in host defense, immunity, and inflammation and even have implications in cancer. Thus, dysregulation of NF-κB activity contributes to numerous autoimmune and inflammatory disease states. With this, the availability of new chemical pathway probes will further enhance understanding of this complex network.

The first project encompassed the synthesis and characterization of three phenotypic probes designed to be selective modulators of the NF-κB signaling pathway in either human embryonic kidney (HEK) 293, HEK 293T or pre-B leukemia 697 cells. The second project focused on two probes that are target-oriented within the NF-κB pathway and are specific modulators of the intracellular signaling protein, nucleotide-binding oligomerization domain-1 (NOD1).

Within each project area first-in-class chemical probes were identified and characterized as new research-tool compounds. The synthetic routes for each probe, including their general physicochemical and pharmacological properties, are summarized in this report.

## Results and Discussion

**Phenotypic screening for noncanonical NF-κB pathway selective inhibitors of IL-8 production in HEK 293, HEK 293T (ML029) and 697 pre-B cells (ML236 and ML237):** Most pathways for NF-κB activation converge on the IκB kinases (IKKs), and more than nine signaling routes have been identified [6]. While IKKs therefore represent attractive targets for drug discovery programs, the selectivity envisioned for an acceptable therapeutic index has remained elusive as inhibitors of IKKs indiscriminately suppress all known NF-κB activation pathways. Within this project, new probes were sought that were not active via the currently known receptor-driven pathways and IκB kinases, but attenuated NF-κB transcriptional activity as measured by a luciferase-based reporter gene assay with validation using a panel of known receptor and kinase-based counter screens [7].

Because activation of NF-κB is known to be initiated through protein kinase C (PKC), we hypothesized that selectivity could be possible by the fact that PKC activation occurs downstream from cell membrane antigen and growth-factor receptors yet is still upstream of IKKγ, potentially by inhibition of a new target protein or novel protein–protein interaction. Using cell-based HTS reporter gene assays, a series of chemical probes were identified that selectively inhibit this unique PKC-induced NF-κB pathway without modulating other NF-κB activation pathways such as those including the cytosolic proteins CARMA1, Bcl-10, MALT1, TRAF6 and Ubc13 [8].

The first probe identified within this series was the benzimidazole ML029 (**4**), which exhibited an IC_50_ of 0.07 μM in the HEK 293 cell assay with corresponding well-defined structure–activity relationships (SAR) through analogue synthesis [9]. This probe was discovered after two separate screening campaigns totaling ~110,000 compounds. At concentrations up to 8 μM, **4** failed to suppress PKCβ and PKCθ (the PKC family members implicated in TCR/BCR signaling) and IKKβ, while known PKC and IKK inhibitors and the broad-spectrum kinase inhibitor staurosporine afforded potent inhibition [9–11]. Further selectivity profiling revealed that **4** inhibited (>50% at 10 uM) only 3 out of the 353 kinases surveyed by using a KINOMEscan™ (DiscoveRx) platform. None of these 3 (TLK1 (70% inhibition), Raf (57%), and JAK2 (53%)) are relevant to NF-κB pathway regulation.

**Scheme 1:**
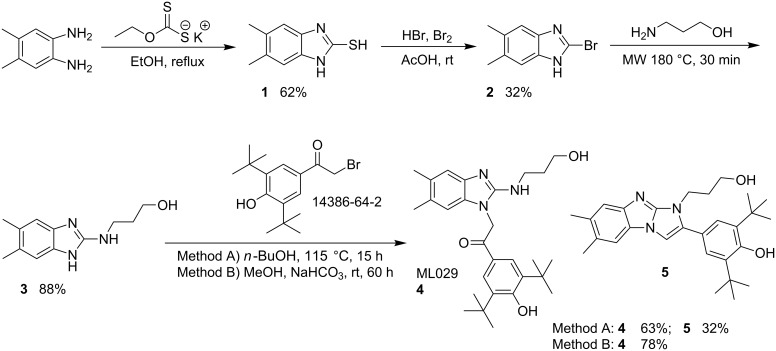
Synthesis of ML029 (**4**).

To prepare **4**, a synthetic route (Scheme 1) was optimized in a manner that allowed for the preparation of related analogues [9]. The intermediate **1** was prepared through condensation of 4,5-dimethylbenzene-1,2-diamine with potassium ethyl xanthate in ethanol under reflux. Bromination of **1** led to the key intermediate 2-bromo-5,6-dimethylbenzimidazole (**2**), which reacted smoothly with 3-aminopropanol to give the amine **3** in high yield and purity after extraction. The solvent-free microwave process employed was superior to traditional oil-bath thermal heating, which tended to generate crude material requiring extensive purification. Ultimately, probe **4** was prepared (Scheme 1, Method A) in moderate yield by reaction of **3** with α-bromo-3,5-di-*tert*-butyl-4-hydroxyacetophenone in *n*-butanol under reflux. Importantly, formation of the tricyclic condensation side product **5** was completely eliminated by performing the reaction with sodium bicarbonate at 23 °C in methanol (Scheme 1, Method B). Thus, compound **4** was isolated as a lyophilized white powder in 78% yield and >98% purity.

Deeper in vitro biological investigation using **4** as a chemical probe showed that it was not active (>30 μM) in more physiologically relevant HEK 293T and 697 pre-B cell lines. Therefore, an HTS campaign was initiated to identify compounds active in these specific cell subtypes by using the established HTS NF-κB reporter gene assay format. This included screening of a larger (~330,600 compound) MLSMR collection to ensure identification of additional chemical scaffolds not previously available. From this campaign two new cell-active NF-κB inhibitors were derived that were both selective against the NF-κB pathway induced by known PKC activators (phorbol myristic acetate (PMA)/ionomycin) and were selective toward the 697 pre-B cell line. It is interesting to note that no compounds meeting probe selectivity criteria were identified within the T cell (HEK 293T) specific assay. The first oxadiazole-based probe ML236 (**8**) was potent (0.035 μM) in the 697 pre-B cell line and >400-fold selective over both the NF-κB activation in HEK 293T cells and against TNFα-mediated NF-κB activation. In comparison, the second oxazole-based probe **12** is also potent (0.2 μM) in the 697 pre-B cell line and >400-fold selective over both the NF-κB activation in HEK 293T cells and against TNFα mediated NF-κB activation as measured in our reporter gene assay format.

**Scheme 2:**
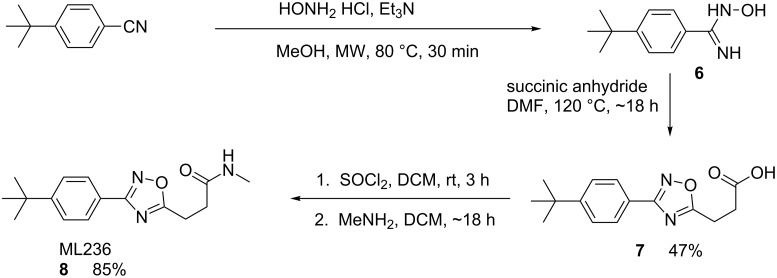
Synthesis of ML236 (**8**).

Probe **8** was synthesized as shown in Scheme 2 [12]. Reaction of 4-*tert*-butyl-cyanobenzene with hydroxylamine hydrochloride under microwave heating conditions resulted in the hydroxyamidine **6**. This intermediate was condensed with succinic anhydride to provide the oxadiazole acid **7**. Conversion of **7** to the corresponding acid chloride and subsequent amidation with excess methylamine afforded **8** in excellent yield after normal phase chromatography. This route was employed to make a variety of analogues and was generally conducted efficiently without purification of any intermediates. This enabled several analogues to be prepared in parallel, using mass-directed reverse-phase preparative HPLC to recover the pure isolates.

**Scheme 3:**
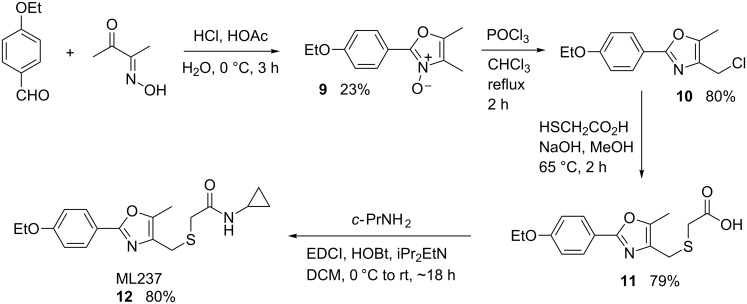
Synthesis of ML237 (**12**).

The second related probe, ML237, (**12**) was synthesized as shown in Scheme 3 [12]. Commercially available 4-ethoxybenzaldehyde was condensed with diacetyl monoxime under acidic conditions. The resulting oxazole *N*-oxide **9** was converted to the chloromethyloxazole **10** via selective chlorination with phosphorous oxychloride in good yield. Addition of thioglycolic acid led to the carboxylic acid **11**, which was coupled to cyclopropylamine to give **12** using a standard amidation protocol. The compound was purified by normal phase chromatography and was recovered as a white solid with a sharply defined melting point. The route was also amenable toward the preparation of related analogues. Generally, **9** was used without purification and the entire route was conducted efficiently without chromatographic purification of intermediates.

As a summary of project one, three novel chemical probes were discovered and optimized within this initiative. Tractable and efficient synthetic routes have been developed and are reported. One HEK 293T and two 697 pre-B cell specific probes are now available as research tools either through the Sanford-Burnham Center or through commercial sources. These probes were identified from a phenotypic screen and thus the precise cellular protein target is yet to be identified. Each probe performs in a noncanonical manner and inhibits NF-κB activity outside of the known, well-characterized pathways [7]. Determination of the precise signaling mechanism is the subject of ongoing research in our laboratories.

**Discovering modulators of NF-κB signaling via selective nucleotide-binding oligomerization domain-1 (NOD1) inhibition (ML130 and ML146):** The second program targeted proteins in the mammalian innate immune system that confer defense by detection of specific microbial ligands, or pathogen-associated molecular pattern microbial sensors known to house NOD1 (nucleotide-binding oligomerization domain-1 protein). Dysregulation of the NOD signaling pathway is also strongly associated with exacerbation of postinfection systemic disease states; however, its upregulation at the gene level can enhance systemic innate immunity [13]. The NOD1 cytosolic bacterial sensor falls within this class of NF-κB transcription pathway activators [14]. The NOD-like receptor (NLR) family reportedly comprises a large number of proteins from both vertebrate and invertebrate animal species, with >20 human proteins recognized [15–21]. Some NLR proteins have been shown to detect bacterial cell-wall components including lipopolysaccharides and/or peptidoglycan (NOD1 or 2) along with those of bacterial flagellin [22–25]. Importantly, the NOD1 protein primarily recognizes Gram-negative bacteria, in contrast to the more widely studied sensor NOD2, which participates in immunity against Gram-positive bacteria. Additionally, NOD1 has been associated with the induction of NF-κB activation, caspase-1 activation and apoptosis [26–28]. Genetic mutations in NOD are associated with numerous inflammatory conditions, including Crohn’s disease and pancreatitis [29–34]. The identification of probes relevant to the NOD family of NLR proteins will improve understanding of the NOD1-mediated signaling pathway and also may translate to the discovery of new therapeutics for inflammatory diseases.

Probes **ML130** (**13**) and **ML146** (**17**) were identified as NOD1-selective molecules from an HTS campaign involving ~290,000 compounds. Compound **13** (IC_50_ = 0.52 μM) and compound **17** (IC_50_ = 1.54 μM) each inhibited γ-tri-DAP-stimulated (a gamma-tri-diaminopimelic acid derivative and NOD1-dependent signaling ligand) luciferase production in HEK 293T cells, which has endogenous NOD1 levels at submicromolar concentration as determined in a NF-κB-linked reporter assay. Both probes selectively (>40-fold for **13**, >8-fold for **17**) inhibited NOD1-dependent activation of the NF-κB pathway without inhibiting MDP-stimulated (muramyl dipeptide and a NOD2-dependent signaling ligand) signal transduction in reporter cell lines containing either low or overexpressed NOD2 proteins. Probes **13** and **17** were also selective over the non-NOD-stimulated NF-κB pathways (TNF-α, doxorubicin and PMA/ionomycin) in these reporter assays. Both of the probes, and their close analogues as discovered through SAR studies, selectively inhibited IL-8 secretion and the biologically relevant terminal effect of NOD1 (γ-tri-DAP) dependent NF-κB activation. Additionally, they neither inhibited NOD2-dependent nor TNF-α-dependent IL-8 secretion in biologically relevant MCF-7 cells. While **13** is the more potent and selective of these probes, **17** also met probe criteria and represents a second *bonafide* scaffold for a NOD1-selective probe.

**Scheme 4:**
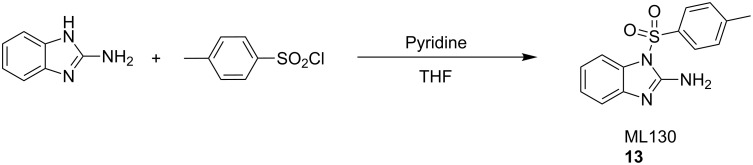
Synthesis of ML130 (**13**).

ML130 (**13**) was synthesized as shown in Scheme 4 [35]. Commercially available 2-aminobenzimidazole was treated with *p*-toluenesulfonyl chloride in the presence of pyridine to obtain **13**. By performing the reaction at high concentration, it was generally simple to purify the reaction mixture directly without work up using normal-phase chromatography.

**Scheme 5:**
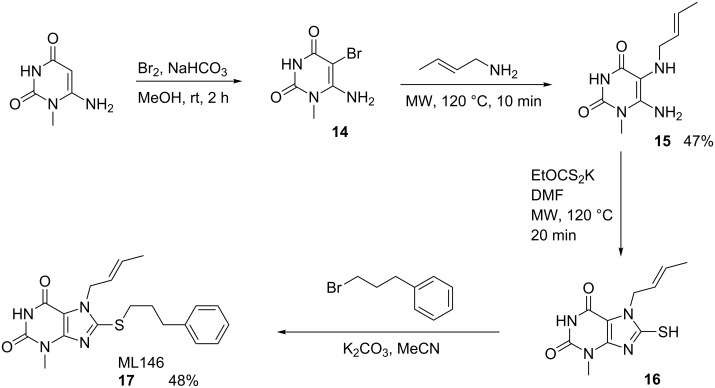
Synthesis of ML146 (**17**).

ML146 (**17**) was synthesized as shown in Scheme 5. Commercially available 6-amino-1-methyluracil was brominated to give **14**, which was reacted without purification with crotylamine under microwave heating conditions to provide **15**. Reaction of **15** with potassium ethyl xanthogenate, again under microwave heating conditions, led to the bicyclic thiol **16**. Without purification, **16** was added to 1-bromo-3-phenylpropane in the presence of potassium carbonate to provide **17**. Several analogues were efficiently prepared without chromatographic purification of intermediates, and the final compounds were purified by normal-phase chromatography if necessary [12].

As a summary of the second project, two novel chemical probes that are selective for NOD1 but not NOD2 signaling, were discovered and optimized within the scope of the program. Tractable and efficient synthetic methods have been employed and are reported. Both NOD1 selective probes are now available as research tools either through the Sanford-Burnham Center or through commercial sources.

## Chemical probe physicochemical and pharmacological properties

Overall, the project probes presented physicochemical properties consistent with those of well-recognized drug-like molecules (Table 1). Molecular weights ranged from 287 to 465 g/mol. Calculated log *P* values were between 3.1 and 3.5 with the exception of **4**, which had an exceptionally high lipophilic value of 7.2 due to the presence of two *tert*-butyl moieties on the phenyl ring. The probes all contain from one to five H-bond donors and acceptors, and polar surface areas ranging from 67 to 90 Å^2^. Based on current hit-to-lead trends within the pharmaceutical industry, all probes discussed provide qualified starting points for advanced lead-optimization programs.

**Table 1:** Calculated properties of probes.

Property	**4**	**8**	**12**	**13**	**17**

Molecular weight [g/mol]	465.6	287.3	346.4	287.3	370.4
Molecular formula	C_28_H_39_N_3_O_3_	C_16_H_21_N_3_O_2_	C_18_H_22_N_2_O_3_S	C_14_H_13_N_3_O_2_S	C_19_H_22_N_4_O_2_S
XLogP3-AA	7.2	3.0	3.1	3.2	3.5
H-Bond donors	3	1	1	1	1
H-Bond acceptors	5	4	4	4	3
Polar surface area [Å^2^]	85.2	68.0	89.7	86.4	67.2
Heavy atom count	34	21	24	20	26

The probes were evaluated using known in vitro ADME/T assays to understand their overall pharmacological properties (Table 2). Probe **4** exhibited good solubility especially as the pH decreased from 6.2 to 5, thereby mitigating the high calculated log *P* to some degree. Probes **8** and **12** also showed good solubility. Probe **13** had moderate solubility, and **17** showed the lowest solubility across the pH range but still approximately equal to its IC_50_ value. The PAMPA (parallel artificial membrane permeability assay) assay is used as an in vitro model of passive, transcellular permeability. An artificial membrane immobilized on a filter is placed between a donor and acceptor compartment. In this test, the probes exhibited good cell permeability overall. Although metabolic stability was poor in the presence of murine microsomes, all five probes were more stable in the presence of human hepatic microsomes. Most notably, **8** showed no apparent instability when subjected to human hepatic microsomes after one hour of exposure. It is not yet known whether it represents a species specific CPY450 inhibitor. Importantly, none of the probes showed evidence of cytotoxicity in immortalized human hepatocyctes (Fa2N-4 cells) and in the NCI-60 cell line cytotoxicity panel with 10 and 50 μM test concentrations [36].

**Table 2:** Summary of in vitro ADME/T properties of probes.

Property	**4**	**8**	**12**	**13**	**17**

Aqueous solubility (μM) pH 5.0/6.2/7.4	90/1.1/1.5	92/101/103	112/133/160	7.0/5.9/7.0	1.5/1.8/1.7
**PAMPA P_e_** (10^−6^ cm/s)^a^ Donor pH: 5.0/6.2/7.4 Acceptor pH: 7.4	299/710/441	751/755/746	513/541/59	491/562/382	1269/1516/1344
Hepatic microsome stability^b^ Human/mouse (% remaining)	26/0.5	100/4.9	22.7/0	41.8/0.8	8.8/0.86
Hepatic toxicity LC_50_ (µM)	>50	>50	>50	>50	>50

^a^Compound at 50 μM (typical PAMPA P_e_ permeability classification: low 5 × 10^−6^, moderate 250 × 10^−6^, high 1000 × 10^−6^); ^b^Compound at 1 μM, 60 min.

## Conclusion

The high-throughput screening of cell-based phenotypic and specific NOD1 protein-based assay targets within the NF-κB pathway has generated a series of unique chemical probes that are now available and useful for investigators to access and utilize in future studies. The compounds are well characterized using in vitro assay panels and are not pathway-associated kinase-family inhibitors. Research efforts are ongoing to improve molecular potency and properties to make each chemical scaffold family suitable for advanced in vivo studies. Each probe represents a low-molecular-weight, “rule of 5” compliant starting point for target identification or lead optimization efforts. In screening the MLSMR, and with subsequent analogue synthesis, we have demonstrated that unique and tractable hits can be identified by using the available NIH-MLSMR compound collection.

## Supporting Information

File 1 Experimental procedures for synthesizing compounds **1**–**4** and **6**–**17**.

File 2 NMR Spectra for ML029 (**4**), ML236 (**8**), ML237 (**12**), ML130 (**13**) and ML146 (**17**).
