# Supplementary material for: Synthesis and physicochemical characterization of novel phenotypic probes targeting the nuclear factor-kappa B signaling pathway
Source: Beilstein J Org Chem. 2013 May 8;9:900–7. doi: 10.3762/bjoc.9.103 (PMC3678512; doi:10.3762/bjoc.9.103)
Supplement: File 1 — Experimental procedures for synthesizing compounds 1–4 and 6–17. [file Beilstein_J_Org_Chem-09-900-s001.pdf]

Supporting Information

for

Synthesis and physicochemical characterization of novel phenotypic probes targeting the nuclear factor-kappa B signaling pathway

Paul M. Hershberger[1], Satyamaheshwar Peddibhotla[1], E. Hampton Sessions[1], Daniela B. Divlianska[1], Ricardo G. Correa[2], Anthony B. Pinkerton[2], John C. Reed[2] and Gregory P. Roth*[1]
Address: [1]Conrad Prebys Center for Chemical Genomics, Sanford-Burnham Medical Research Institute at Lake Nona, 6400 Sanger Road Orlando, FL 32827, USA and [2]Sanford-Burnham Medical Research Institute, 10901 North Torrey Pines Road, La Jolla, CA 92037, USA

Email: Gregory P. Roth - groth@sanfordburnham.org
*Corresponding author

Experimental procedures for synthesizing compounds **1–4** and **6–17**.

General experimental procedures. All reactions involving air- and moisture-sensitive reagents and solvents were performed under a nitrogen atmosphere using standard chemical techniques. Anhydrous solvents were purchased and used fresh from Sigma-Aldrich or EMD biosciences. All organic reagents were used as purchased. Analytical thin-layer chromatography was performed on Partisil K6F silica gel 60 Å, 250 μm. Preparative TLC was performed using Partisil® PKL5F silica gel plate (150 Å, 20 × 20, 1000 μm) and developed with an appropriate solvent. Bands were identified by UV, and the silica was removed from the plate and placed into 20 mL vials. Organic residues were extracted by suspending the silica gel in 5:95 methanol/ethyl acetate. This was followed by two more elutions with 5:95 methanol/ethyl acetate. The combined washings were evaporated and dissolved in dichloromethane and filtered through GELMAN® NYLON ACRODISC® (0.45 μm) to remove fine particles of silica gel. Microwave-assisted reactions were performed using a CEM Discover system. All NMR spectra for the synthetic materials were recorded on a Bruker Avance II 400 or Avance

III 500 MHz instrument. ^{1}H and ^{13}C chemical shifts are reported in δ values in ppm in the corresponding solvent. All solvents used for chromatography on the synthetic materials were Fisher Chemical HPLC grade, and the water was Millipore Milli-Q PP filtered. High-resolution mass spectrometry (HRMS) was carried out on an Agilent 6224A LC/MS–TOF instrument in the ESI–TOF positive mode. LC/MS analysis and preparative separations of synthetic materials was conducted on a Waters Inc. preparative AutoPurification system, which consists of a 2767 sample manager, a 2545 binary gradient module, a system fluidics organizer, a 2489 UV–vis detector, and a 3100 mass detector, all controlled with MassLynx software. A Sunfire Analytical C18 5 µm column (4.6 × 50 mm) and a stepwise gradient {10% [(MeCN + 0.1% TFA) in (water + 0.1% TFA)] for 1min.; 10–98% for 3 min and 98% for 2 min} was used for analytical LC/MS. All tested final compounds were determined to be ≥95% pure by HPLC-UV/MS & ^{1}H NMR.

5,6-Dimethyl-1*H*-benzo[*d*]imidazole-2-thiol (**1**): A 500 mL round bottom flask was charged with 4,5-dimethylbenzene-1,2-diamine (10 g, 73.4 mmol), potassium ethyl xanthate (16 g, 99.8 mmol), 100 mL ethanol and 14 mL water. The mixture was heated under reflux for 3 h. Norit-A decolorizing charcoal (~ 1 g) was added and mixture was heated under reflux for an additional 10 minutes before filtering over celite. The filtrate was heated to 60–70 °C, and 100 mL water followed by 50% acetic acid solution (8 mL) was added slowly. The suspension was cooled at 4 °C for 3 h and the solids were filtered and dried under vacuum to afford the 2-mercapto-5,6-dimethylbenzimidazole (**1,** 8.1 g, 62%) as a tan solid. ^{1}H NMR (400 MHz, CD_3OD) δ 6.99 (s, 2H), 2.30 (s, 6H). ESI–MS (*m/z*): [M + H] calcd for $C_9H_{10}N_2S$ = 179.1, found = 179.1.

2-Bromo-5,6-dimethyl-1*H*-benzo[*d*]imidazole (**2**): To a 250 mL round-bottom flask equipped with a mechanical stirrer was added 20 mL acetic acid and 2.1 mL of 48% aqueous HBr solution and the mixture was cooled to 0 °C over an ice bath. **1** (2.5 g, 14 mmol) was added followed by dropwise addition of bromine (2.6 mL, 50 mmol) with medium stirring over 10 min. After about half of the bromine was added, the mixture turned bright orange and became thick such that vigorous stirring was needed to disperse the solids. After all the bromine had been added, 40 mL acetic acid was added, and the mixture was stirred at 23 °C for 4.5 h. The mixture was then diluted with 45 mL of water and cooled to 0 °C over an ice bath. Solid NaOH was added to adjust the pH of the solution to **4**. The clear solution was cooled at 4 °C for 3 days during which the 2-bromobenzimidazole **2** precipitated as a light brown solid that was filtered, washed extensively with ice-cold water and air-dried (1.02 g, 32%). ^{1}H NMR (400 MHz, DMSO- d_6) δ 12.92 (s, 1H), 7.27 (s, 2H), 2.27 (s, 6H); ESI–MS (*m/z*): Calcd for $C_9H_9BrN_2$ [M + H] = 225.0 and [M + 2 + H] = 227.0, found [M + H] = 224.8 and [M + 2 + H] = 226.7.

3-((5,6-Dimethyl-1*H*-benzo[*d*]imidazol-2-yl)amino)propan-1-ol (**3**): The 2-bromobenzimidazole **2** (0.5 g, 2.23 mmol) was combined with 3-aminopropanol (1 mL, 13 mmol) in a 35 mL microwave reaction tube. The dark orange solution was heated by microwave for 30 min at 180 °C. The mixture was then diluted with 20 mL ethyl acetate and extracted with saturated $NaHCO_3$ solution (3 × 5 mL). The organic layer was dried

over anhydrous $MgSO_4$ and concentrated to a brown residue, which was triturated with diethyl ether (3 × 10 mL). The ether wash was discarded and the residual solid was dried under vacuum to afford **3** (0.42 g, 88%), which was used without further purification. ^{1}H NMR (400 MHz, CD_3OD) δ 6.96 (s, 2H), 3.67 (t, *J* = 6.2 Hz, 2H), 3.42 (t, *J* = 6.8 Hz, 2H), 2.26 (s, 6H), 1.84 (p, *J* = 6.5 Hz, 2H); ESI–MS (*m/z*): [M + H] calcd for $C_{12}H_{18}N_3O$ = 220.1, found = 220.1.

1-(3,5-Di-*tert*-butyl-4-hydroxyphenyl)-2-(2-((3-hydroxypropyl)amino)-5,6-dimethyl-1*H*-benzo[*d*]imidazol-1-yl)ethanone (**4**): A 20 dram vial was charged with 2-aminobenzimidazole **3** (0.37 g, 1.6 mmol), α-bromo-3,5-di-*tert*-butyl-4-hydroxyacetophenone (0.62 g, 1.9 mmol), 10 mL methanol and solid $NaHCO_3$ (3 equiv). The mixture was stirred at 23 °C for 3 days after which the solvent was evaporated and the residue purified by silica-gel flash column chromatography (hexanes to 1:4 hexanes/ethyl acetate) to furnish a yellow film. Lyophilization from a 2:8 acetone/water mixture afforded **4** as a white solid (0.61 g, 78%). ^{1}H NMR (400 MHz, acetone-d_6) δ 7.87 (s, 2H), 6.95 (s, 1H), 6.77 (s, 1H), 5.94 (s, 1H), 5.52 (s, 2H), 3.55-3.47 (m, 2H), 3.42 (t, *J* = 5.6 Hz, 2H), 2.14 (s, 3H), 2.12 (s, 3H), 1.56 (p, *J* = 6.0 Hz, 2H), 1.40 (s, 18H); ^{13}C NMR (100 MHz, acetone-d_6-) δ 191.58, 159.66, 156.73, 141.17, 137.58, 134.43, 128.85, 127.29, 127.19, 126.16, 116.74, 108.72, 57.65, 48.47, 39.19, 34.90, 34.67, 19.77, 19.74; ESI–MS (*m/z*): [M + H] calcd for $C_{28}H_{39}N_3O_3$ = 466.3, found [M + H] = 466.1; HRMS–ESI: [M + H] calcd for $C_{28}H_{39}N_3O_3$ = 466.3070, found = 466.3091.

4-(*tert*-Butyl)-*N*-hydroxybenzimidamide (**6**): A microwave vessel was charged with 4-*tert*-butylbenzonitrile (1.06 g, 6.66 mmol) and methanol (10 mL). Triethylamine (1.03 mL, 6.66 mmol) and hydroxylamine hydrochloride (460.0 mg, 6.66 mmol) were added. The mixture was heated in the microwave system at 80 °C for 30 minutes. The mixture was transferred to a round bottom flask and the solvent was removed. The flask was placed under high vacuum for 30 minutes. The solid product, **6**, was used without purification.

3-(3-(4-(*tert*-Butyl)phenyl)-1,2,4-oxadiazol-5-yl)propanoic acid (**7**): The crude solid **6** was dissolved in *N, N'*-dimethylformamide (10 mL). Succinic anhydride (880.0 mg, 6.66 mmol) was added. The mixture was stirred at 120 °C overnight. The solution was cooled to room temperature and treated with 60 mL of 1 N HCl. The mixture was extracted with ethyl acetate (3 × 40 mL), washed with saturated brine (60 mL), dried over anhydrous sodium sulfate, filtered and concentrated. The crude product was triturated with hexanes (100 mL) and filtered. The white solid **7** (850.0 mg, 47% for two steps) was used in the next step without further purification. ^{1}H NMR (400 MHz, $CDCl_3$) δ 7.97 (d, *J* = 10.5 Hz, 2H), 7.49 (d, *J* = 10.3 Hz, 2H), 3.28 (t, *J* = 7.4 Hz, 2H), 3.05 (t, *J* = 7.4 Hz, 2H), 1.35 (s, 9H). ESI–MS (*m/z*): [M + H] = 275.

3-(3-(4-(*tert*-Butyl)phenyl)-1,2,4-oxadiazol-5-yl)-*N*-methylpropanamide (**8**): A round-bottom flask was charged with **7** (314.0 mg, 1.24 mmol) and dichloromethane (10.0 mL). The contents were chilled to 0 °C under an inert atmosphere. Thionyl chloride (0.18 mL, 2.48 mmol, 2.0 equiv) was added dropwise. The resulting solution

was stirred for 30 minutes at 0 °C. The mixture was then stirred at room temperature for 3 h. The solvent was removed under reduced pressure and the flask was put under high vacuum for 30 minutes. The solid acid chloride was dissolved in methylene chloride (10 mL) at 0 °C under N_2. A methylamine solution (2 M solution in tetrahydrofuran, 1.3 mL, 2.48 mmol, 2.0 equiv) was added dropwise. The mixture was stirred for 1 hour at 0 °C. The solution was diluted with dichloromethane (30 mL) and was washed with saturated ammonium chloride solution (30 mL). The organic and aqueous layers were separated. The aqueous layer was extracted with dichloromethane (2 × 40 mL). The combined organic layer was washed with saturated brine (30 mL), dried over anhydrous sodium sulfate, filtered and concentrated under reduced pressure. The crude product was purified by column chromatography eluting with 30–50% ethyl acetate/hexanes to afford **8** as a white solid (302.9 mg, 85% yield): ^{1}H NMR (400 MHz, $CDCl_3$) δ 7.98 (d, *J* = 10.5 Hz, 2H), 7.53 (d, *J* = 10.3 Hz, 2H), 5.77 (br s, 1H), 3.32 (t, *J* = 7.4 Hz, 2H), 2.86 (d, *J* = 4.9 Hz, 3H), 2.79 (t, *J* = 7.4 Hz, 2H), 1.37 (s, 9H); ^{13}C (100 MHz, $CDCl_3$) δ 178.7, 170.9, 168.2, 154.6, 127.2, 125.8, 123.8, 34.9, 32.3, 31.2, 26.5, 22.5; melting point: 120–122 °C. HRMS–ESI: [M + H] calcd for $C_{16}H_{22}N_3O_2$ = 288.1707, found = 288.1694.

4,5-Dimethyl-2-(4-ethoxyphenyl)-oxazole-3-oxide (**9**): A solution of 2,3-butanedione monooxime (5.80 g, 58.3 mmol) and 4-ethoxybenzaldehyde (9.30 g, 62.0 mmol) in acetic acid (50 mL) was cooled to 0 °C before adding 4 M HCl dioxane solution (24 mL, 96.0 mmol). The mixture was stirred at 0 °C for 3 h. Diethyl ether (100 mL) was added and the resulting slurry was stirred for 1 h at 0 °C. The white precipitate was filtered and washed with ether (20 mL). The solid was transferred to a beaker and was treated with water (100 mL) and concentrated ammonia hydroxide solution (12 mL). The mixture was extracted with $CHCl_3$ (2 × 100 mL), washed with saturated brine (100 mL), dried over anhydrous sodium sulfate, filtered and concentrated to afford **9** as a white solid (2.8 g, 23% yield). The compound was used immediately in the next step without further purification.

4-(Chloromethyl)-2-(4-ethoxyphenyl)-5-methyloxazole (**10**): A solution of **9** (2.8 g, 12.0 mmol) in anhydrous $CHCl_3$ (20 mL) was treated with $POCl_3$ (1.2 mL, 13.2 mmol) at room temperature. The mixture was heated under reflux for 2 h, cooled to room temperature, poured into ice water (100 mL), and neutralized with saturated $NaHCO_3$ solution (100 mL). The mixture was extracted with $CHCl_3$ (2 × 100 mL), washed with saturated brine, dried over anhydrous sodium sulfate, filtered and concentrated. The crude product was dissolved in hot hexanes (100 mL), decanted from dark insoluble material and cooled to 0 °C. The resulting solid was filtered and dried to give partially pure **10**, 2.40 g (80%). ^{1}H NMR (400 MHz, $CDCl_3$) δ 8.06 (d, *J* = 8.8 Hz, 1H), 6.99 (d, *J* = 8.9 Hz, 1H), 4.64 (s, 2H), 4.12 (q, *J* = 6.9 Hz, 2H), 2.47 (s, 3H), 1.47 (t, *J* = 6.9 Hz, 3H); ESI–MS (*m/z*): [M + H] = 252.

2-(((2-(4-Ethoxyphenyl)-5-methyloxazol-4-yl)methyl)thio)acetic acid (**11**): A solution of NaOH (770.0 mg, 19.2 mmol) and thioglycolic acid (883.0 mg, 9.6 mmol) in MeOH (30 mL) was stirred at room temperature for 30 minutes. **10** (2.40 g, 9.6 mmol) was added and the mixture was stirred at 65 °C for 2 h. The solvent was removed and the residue was treated with 100 mL of 1 N HCl. The mixture was extracted with ethyl

acetate (2 × 80 mL), washed with saturated brine, dried over anhydrous sodium sulfate, filtered and concentrated. The crude product was triturated with hexanes (100 mL). The solid was filtered and dried to give partially pure **11**, 2.3 g (79%). ^{1}H NMR (500 MHz, $CDCl_3$) δ 7.98 (d, *J* = 8.8 Hz, 2H), 7.00 (d, *J* = 8.9 Hz, 2H), 4.13 (q, *J* = 6.9 Hz, 2H), 3.83 (s, 2H), 3.45 (s, 2H), 2.43 (s, 3H), 1.48 (t, *J* = 6.9 Hz, 3H); ESI–MS (*m/z*): [M + H] = 308.

***N*-Cyclopropyl-2-(((2-(4-ethoxyphenyl)-5-methyloxazol-4-yl)methyl)thio)acetamide** (**12**): A round-bottom flask was charged with **11** (270.0 mg, 0.88 mmol) and dichloromethane (6.0 mL) at 0 °C. *N*,*N*-Diisopropylethylamine (0.31 mL, 1.76 mmol, 2.0 equiv) was added followed by 1-ethyl-3-(3-dimethylaminopropyl) carbodiimide hydrochloride (260.0 mg, 1.32 mmol, 1.50 equiv) and hydroxybenzotriazole (180.0 mg, 1.32 mmol, 1.50 equiv). The resulting solution was stirred for 30 minutes at 0 °C. Cyclopropylamine (0.12 mL, 1.76 mmol, 2.0 equiv) was added and the solution was stirred at room temperature overnight. The solution was diluted with dichloromethane (30 mL) and washed with saturated ammonium chloride solution (30 mL). The organic and aqueous layers were separated. The aqueous layer was extracted with dichloromethane (2 × 30 mL). The combined organics were washed with saturated brine (30 mL), dried over anhydrous sodium sulfate, filtered and concentrated. The crude product was purified by column chromatography, eluting with 30–50% ethyl acetate/hexanes to afford **12** as a white solid (243.4 mg, 80%): ^{1}H NMR (400 MHz, $CDCl_3$) δ 7.89 (d, *J* = 8.9 Hz, 2H), 7.45 (broad s, 1H), 6.95 (d, *J* = 8.9 Hz, 2H), 4.09 (q, *J* = 6.9 Hz, 2H), 3.58 (s, 2H), 3.13 (s, 2H), 2.63 (m, 1H), 2.33 (s, 3H), 1.42 (t, J = 6.9 Hz, 3H), 0.71 (m, 2H), 0.43 (m, 2H);^{13}C NMR (100 MHz, $CDCl_3$) δ 170.6, 160.9, 160.4, 145.1, 132.1, 127.8, 120.1, 114.8, 63.8, 35.4, 27.6, 22.9, 14.9, 10.3, 6.5; melting point: 125–126 °C; HRMS–ESI: [M + H] calcd for $C_{18}H_{23}N_2O_3S$ = 347.1424, found = 347.1414.

2-Amino-1-tosyl-1*H*-benzo[*d*]imidazole (**13**) [1]: A round bottom flask was charged with 2-aminobenzimidazole (100.0 mg, 0.75 mmol) and pyridine (0.31 mL) at room temperature. p-Toluenesulfonyl chloride (147.5 mg, 0.77 mmol, 1.03 equiv) was added in one portion and the resulting cloudy solution was stirred overnight (~12h). Tetrahydrofuran (0.5 mL) was added to facilitate loading of the crude, thick mass onto a preparatory TLC plate, which was subsequently eluted with neat ethyl acetate. The product 13 was removed from silica gel using 10% MeOH ethyl acetate and the product was recovered as a tan solid (75.5 mg, 35% yield). 1H NMR (400 MHz, DMSO-d_6) δ 8.02–7.86 (m, 2H), 7.65 (dt, *J* = 8.0, 0.9 Hz, 1H), 7.50–7.39 (m, 2H), 7.19–7.07 (m, 4H), 7.07–6.96 (m, 1H), 2.34 (d, *J* = 7.1 Hz, 3H); ^{13}C (100 MHz, DMSO-d_6) δ152.2, 146.4, 142.8, 133.8, 130.5, 130.1, 126.8, 124.7, 120.6, 116.0, 112.2, 21.2; melting point: 191–192 °C (with decomposition). HRMS–ESI: [M + H] calcd for $C_{14}H_{13}N_3O_2S$ = 288.0806, found = 288.0803.

6-Amino-5-bromo-1-methylpyrimidine-2,4(1*H*,3*H*)-dione (**14**): Bromine (183 μL, 3.5 mmol) was added to a suspension of 6-amino-1-methyluracil (500.0 mg, 3.5 mmol) and sodium bicarbonate (298 mg, 3.5 mmol) in 20 mL of methanol with vigorous stirring at 0 °C. After stirring for 2 h at room temperature, the mixture was chilled to 4 °C and a white precipitate was collected, stirred in 10 mL of water at 4 °C, recollected, washed with cold water and dried under high vacuum to provide 450 mg (58%) of **14**, which was

used without purification. ^{1}H NMR (partial data, 500 MHz, DMSO-d_6) δ 10.89 (s, 2H), 3.33 (s, 3H).

(*E*)-6-Amino-5-(but-2-en-1-ylamino)-1-methylpyrimidine-2,4(1*H*,3*H*)-dione (**15**): **14** (100 mg, 0.45 mmol) was combined with crotylamine (0.25 mL, 2.73 mmol) and the mixture was heated in the microwave at 120 °C for 10 minutes. After cooling to room temperature, addition of 5 mL of diethyl ether promoted precipitation of a solid, which was washed with 10 mL of diethyl ether to return **15** (45 mg, 47%) as an off-white solid. The material was carried forward without purification.

(*E*)-7-(But-2-en-1-yl)-8-mercapto-3-methyl-1*H*-purine-2,6(3*H*,7*H*)-dione (**16**): A mixture of **15** (100.0 mg, 0.48 mmol), potassium ethyl xanthogenate (305 mg, 1.9 mmol), and anhydrous DMF (2 mL) was heated in the microwave at 120 °C for 20 minutes. After cooling to room temperature, addition of 5 mL of water gave a homogenous solution which was acidified to pH 4–5 with 2 N HCl. The resulting precipitate was collected, washed with cold water and diethyl ether, and dried to return 80.0 mg of **16** as an off-white solid which was used without further purification.

(*E*)-7-(But-2-en-1-yl)-3-methyl-8-((3-phenylpropyl)thio)-1*H*-purine-2,6(3*H*,7*H*)-dione (**17**): **16** (80.0 mg, 0.32 mmol) was dissolved in 2 mL of anhydrous acetonitrile before charging with 1-bromo-3-phenylpropane (107.3 mg, 0.54 mmol) and potassium carbonate (74.5 mg, 0.54 mmol). After stirring at room temperature for 12 h, the solids were filtered, and the filtrate was concentrated to a residue, which was purified by column chromatography, eluting with 30% ethyl acetate/hexanes to provide 56.5 mg (48%) of **17**. ^{1}H NMR (400 MHz, DMSO-d_6) δ 11.03 (s, 1H), 7.26–7.14 (m, 5H), 5.54–5.51 (m, 2H), 4.68–4.67 (m, 2H), 3.26 (s, 3H), 3.19 (t, J = 8 Hz, 2H), 2.67 (t, J = 8 Hz, 2H), 1.96 (quint, J = 8 Hz, 2H), 1.58 (d, J = 4 Hz,3H); ^{13}C NMR (125 MHz, DMSO-d_6) δ 153.76, 150.65, 149.62, 149.45, 140.89, 129.46, 128.31, 125.89, 124.96, 124.19, 107.54, 46.55, 33.79, 31.69, 30.70, 28.42, 17.29. HRMS–ESI: [M + H] for $C_{19}H_{23}N_4O_2S$ = 371.1536, found = 371.1543.

Reference

1. Khan, P.; Correa, R. G.; Divlianska, D. B.; Peddibhotla, S.; Sessions, E. H.; Magnuson, G.; Brown, B.; Yuan, H.; Mangravita-Novo, A.; Vicchiarelli, M.; Su, Y.; Vasile, S.; Smith, L. H.; Diaz, P. W.; Reed, J. C.; Roth, G. P. *ACS Med. Chem. Lett.* **2011,** *2,* 780–785. doi:10.1021/ml200158b
