# Supplementary material for: Synthesis and physicochemical characterization of novel phenotypic probes targeting the nuclear factor-kappa B signaling pathway
Source: Beilstein J Org Chem. 2013 May 8;9:900–7. doi: 10.3762/bjoc.9.103 (PMC3678512; doi:10.3762/bjoc.9.103)

Supporting Information

for

Synthesis and physicochemical characterization of novel phenotypic probes targeting the nuclear factor-kappa B signaling pathway

Paul M. Hershberger[1], Satyamaheshwar Peddibhotla[1], E. Hampton Sessions[1], Daniela B. Divlianska[1], Ricardo G. Correa[2], Anthony B. Pinkerton[2], John C. Reed[2] and Gregory P. Roth*[1]

Address: [1]Conrad Prebys Center for Chemical Genomics, Sanford-Burnham Medical Research Institute at Lake Nona, 6400 Sanger Road Orlando, FL 32827, USA and [2]Sanford-Burnham Medical Research Institute, 10901 North Torrey Pines Road, La Jolla, CA 92037, USA

Email: Gregory P. Roth - groth@sanfordburnham.org

*Corresponding author

NMR Spectra for ML029 (**4**), ML236 (**8**), ML237 (**12**), ML130 (**13**) and ML146 (**17**).

ML029 (**4**). ^{1}H NMR and ^{13}C NMR spectra.

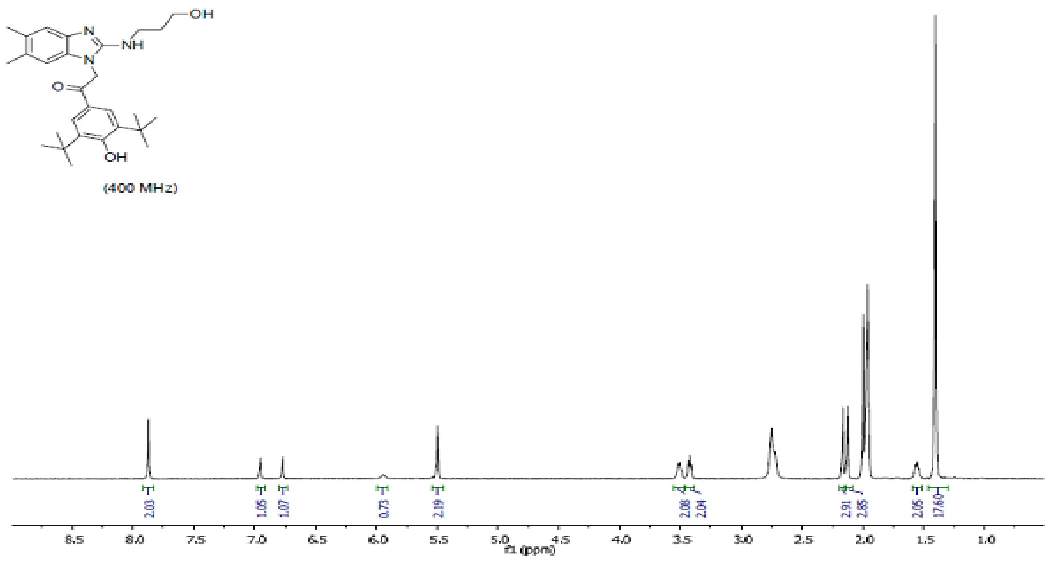

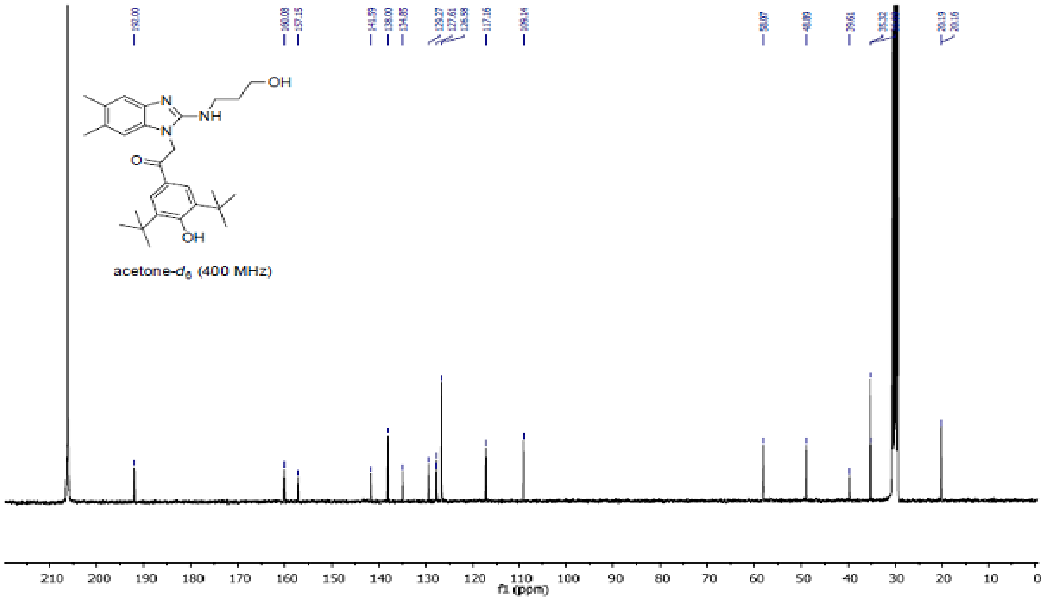

ML236 (**8**). ^{1}H NMR, ^{13}C NMR spectra.

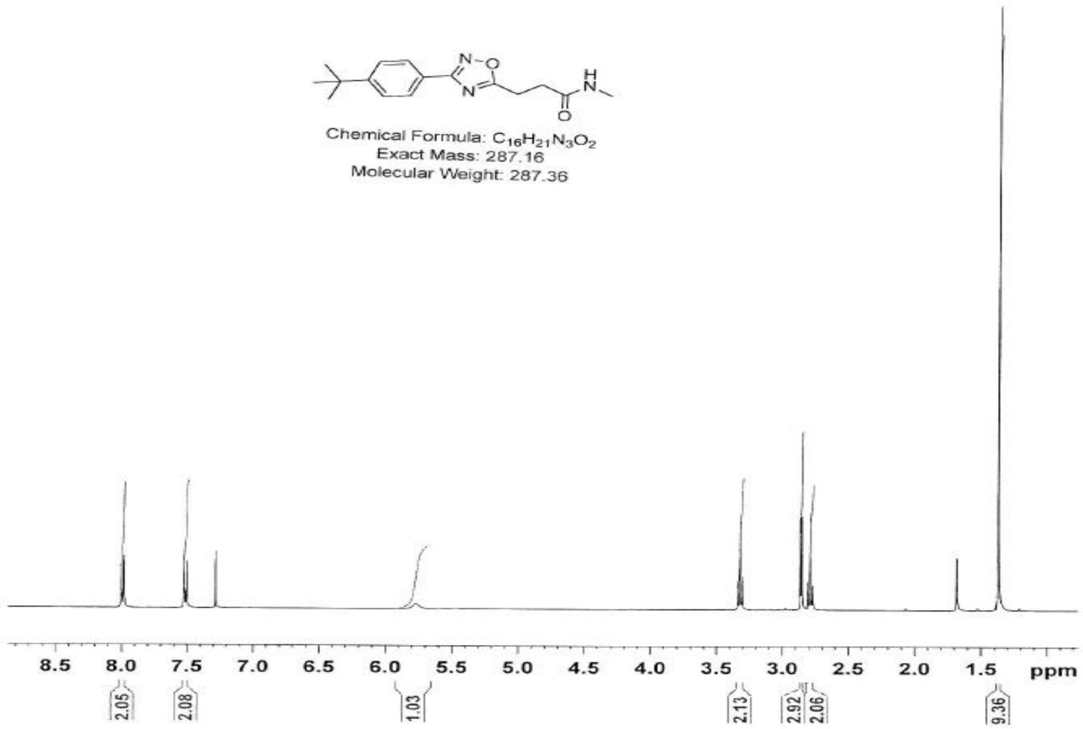

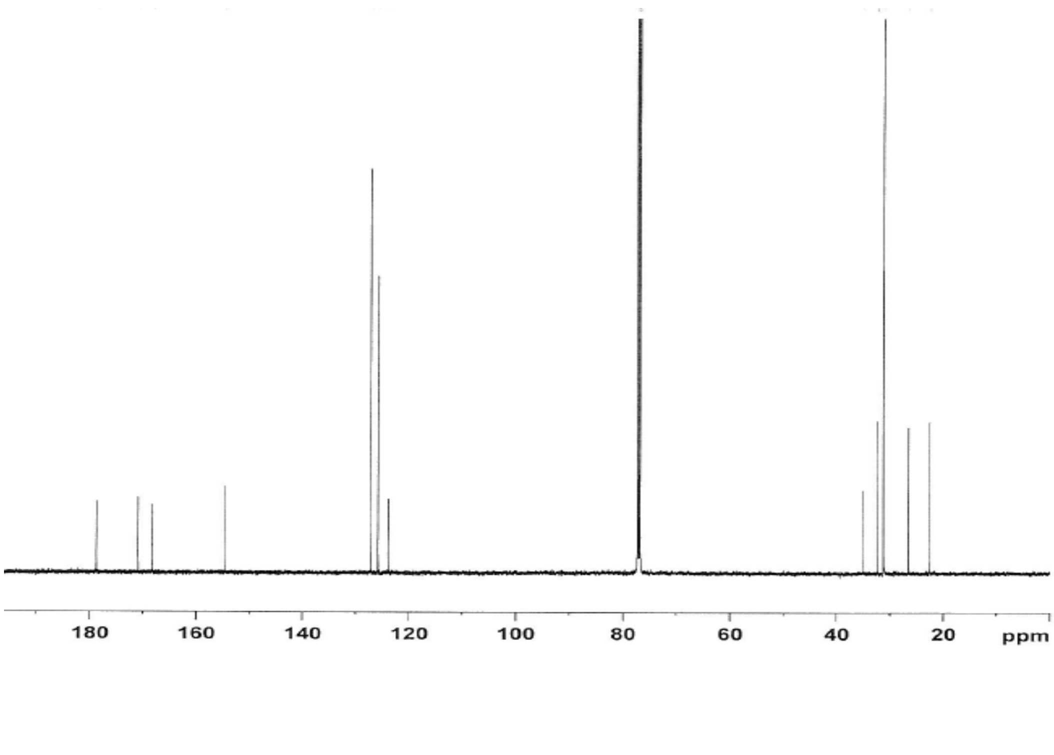

ML237 (**12**). ^{1}H NMR and ^{13}C NMR spectra.

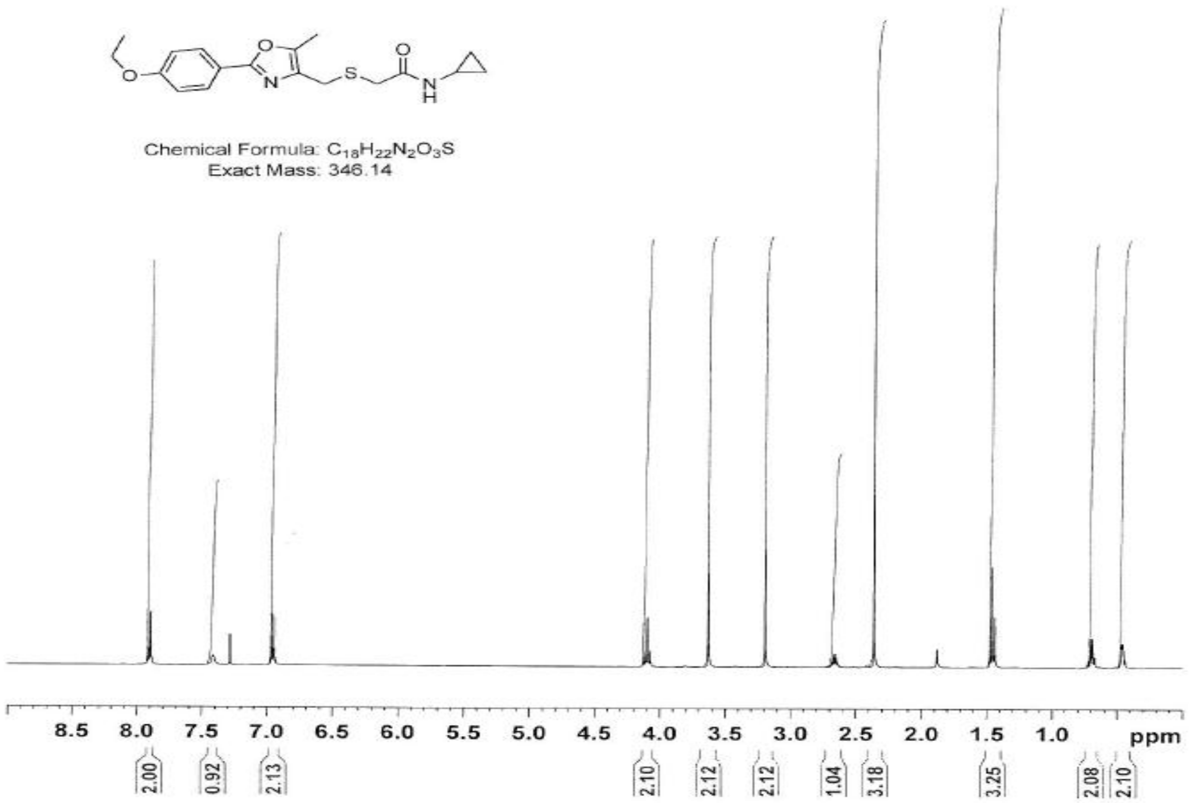

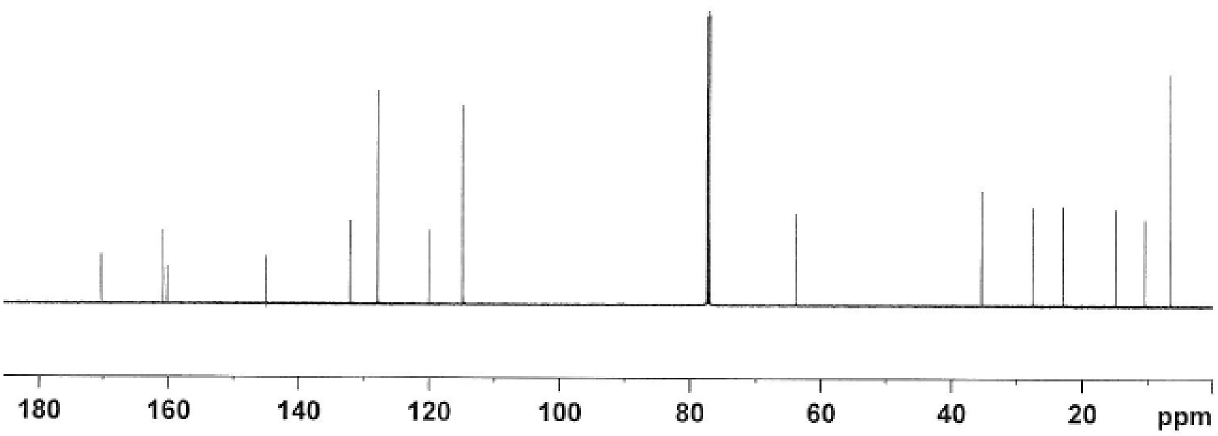

ML130 (**13**). ^{1}H NMR and ^{13}C NMR spectra.

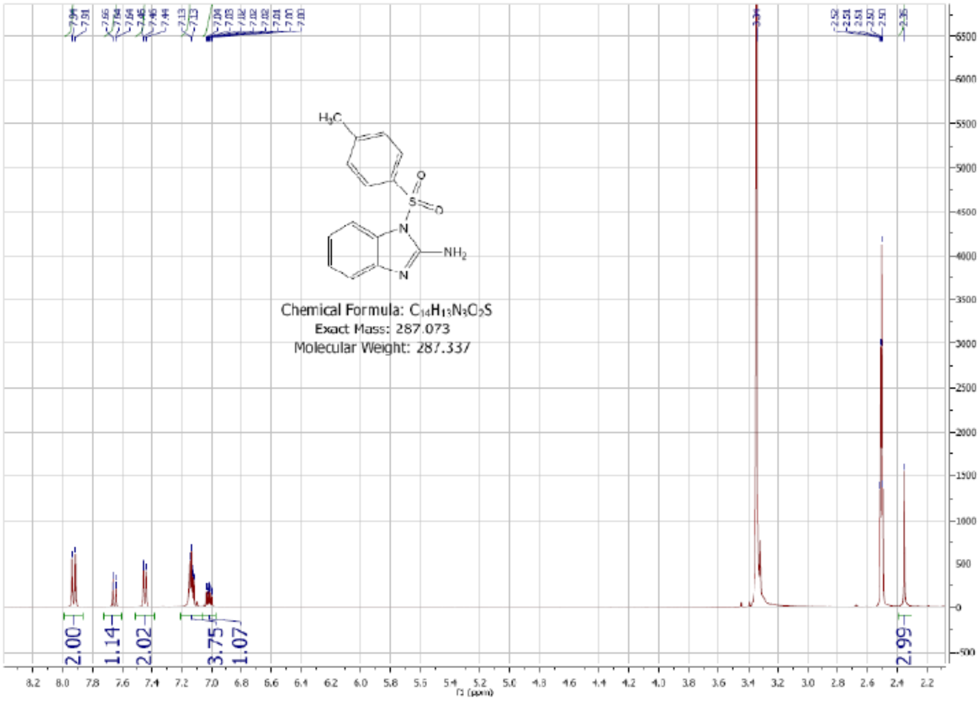

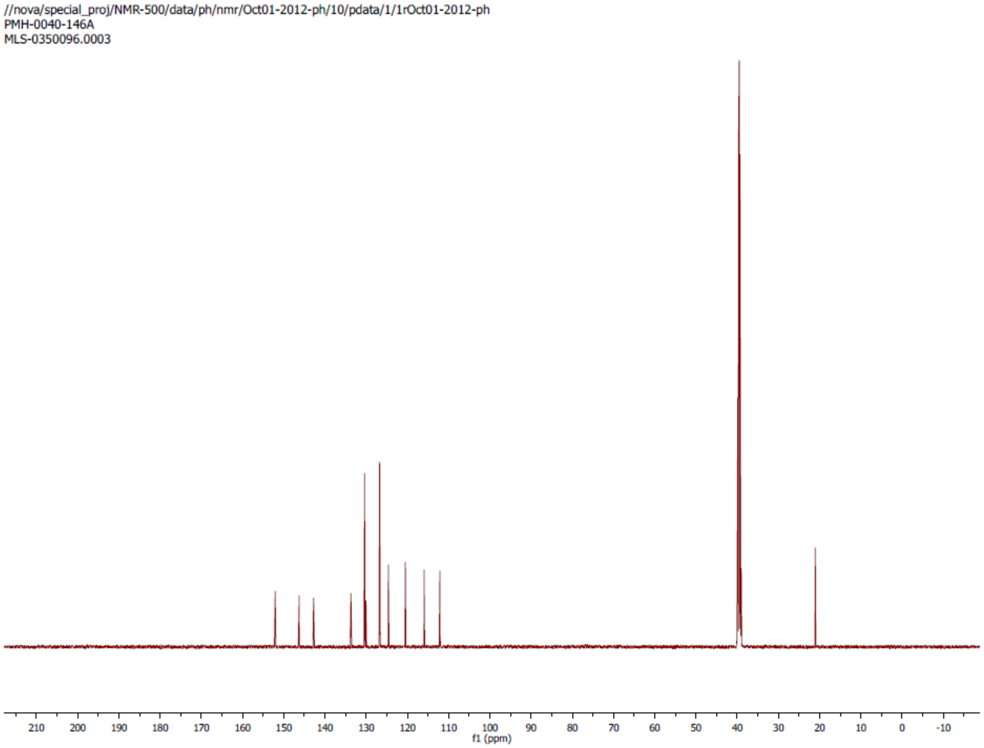

ML146 (**17**). ^{1}H NMR and ^{13}C NMR spectra.

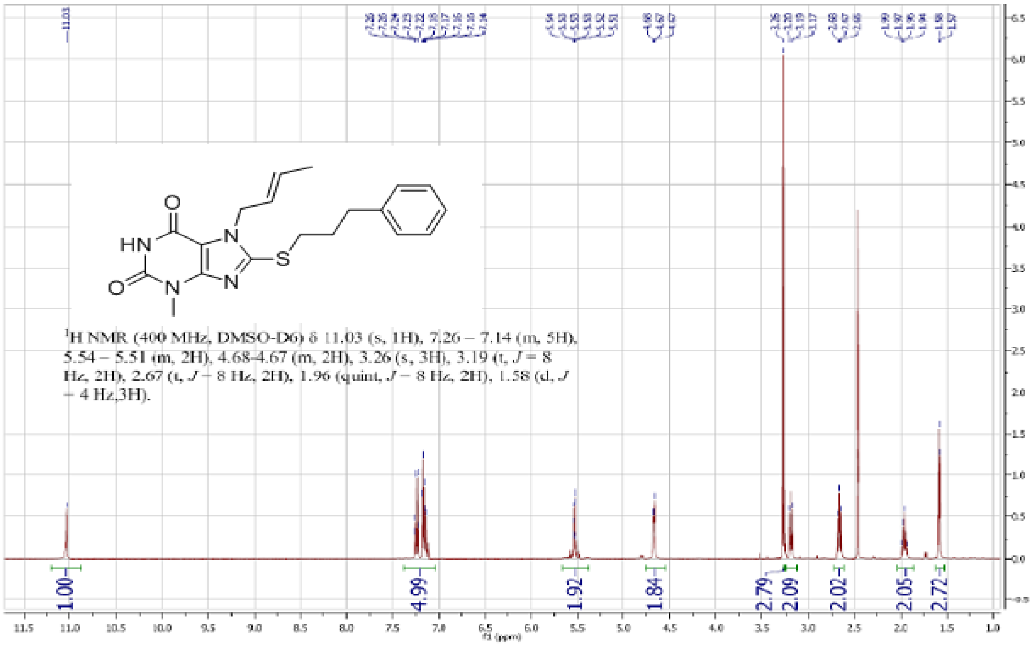

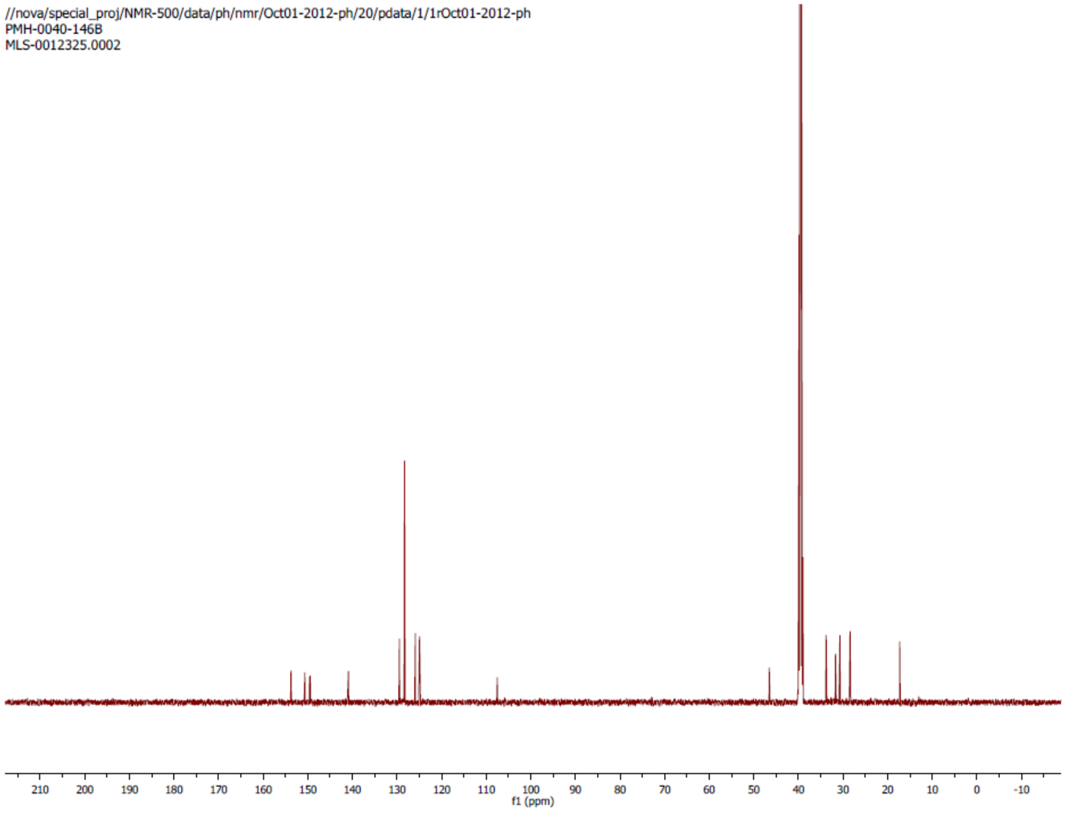

Supplement: File 2 — NMR Spectra for ML029 (4), ML236 (8), ML237 (12), ML130 (13) and ML146 (17). [file Beilstein_J_Org_Chem-09-900-s002.pdf]
